# Development and Validation of Neuro-Cognitive Assessment Battery for Stroke Patients (NCABS) in Pakistan

**DOI:** 10.12669/pjms.345.15083

**Published:** 2018

**Authors:** Syeda Namrah Mahmood, Umaiza Bashir

**Affiliations:** 1 Syeda Namrah Mahmood, MS Clinical Psychology, Institute of Clinical Psychology University of Management and Technology, Lahore, Pakistan; 2 Umaiza Bashir, MS Clinical Psychology, Institute of Clinical Psychology University of Management and Technology, Lahore, Pakistan

**Keywords:** Neuro-cognitive assessment battery, Post stroke cognitive impairment, Unilateral stroke

## Abstract

**Background & Objective:**

Post Stroke Cognitive Impairments (PSCI) occur frequently in stroke survivors resulting in devastating consequences affecting daily living of survivors. Currently, there exists scarcity of sound assessment tools for the evaluation of PSCI as most of the commonly used scales offer a number of clinical (requires motor and linguistic skills) and cultural limitations (requires academic exposure). Therefore, current study was aimed at evaluating the efficacy of Neuro-Cognitive Assessment Battery for Stroke patients (N-CABS) in Pakistan.

**Methods:**

The participants were randomized in two groups including clinical and non-clinical group. N-CABS was administered on 61 clinical and 60 non-clinical participants (mean age=45 years; SD=5.55). Both groups were matched on all demographic variables including; age, gender, education and occupation. The psychometric properties of N-CABS were established through a number of robust measures of validity (construct, concurrent and discriminant validity) and reliability (internal consistency and test retest reliability).

**Results:**

The factor analysis suggested a two-factor solution (labeled as Verbal Cognitive Abilities and Visual Cognitive Abilities) for N-CABS explaining 67% of total variance. A significant test-retest reliability was found (α = 0.92, ^***^p<0.001). The findings of concurrent validity recommended a significant correlation between N-CABS and Montreal Cognitive Assessment (MoCA) (α = 0.82, ^***^p<0.001). Similarly, discriminant validity also revealed significant group differences to exist on N-CABS (^***^p<0.001) suggesting N-CABS be able to discriminate between patients and healthy controls.

**Conclusion:**

The results of the current study favor N-CABS as a psychometrically strong screening instrument to screen PSCI in our culture.

## INTRODUCTION

In only a few years, stroke has appeared as a standalone core Non-Communicable Disease (NCD) and gained recognition as having a larger influence on global health, which was previously rooted under the term of “cardiovascular diseases”.^1^ Pakistan Health and Research Council (PHRC) indicated that the approximate estimation of annual incidents of stroke in Pakistan is 250/100,000, which means almost 350,000 new cases each year.^2^ It has also been criticized that the survey conducted by PMRC lacked proper designing to assess neurological conditions including stroke.^3^ The major reason for the scarcity of epidemiological data on stroke is unavailability of validated screening scales for neurological diseases in native language.^3^

The term stroke related disability is not only limited to physical health but it also covers the major aspect of mental health as well, that is cognition.^4^ Approximately 20 to 80% of the stroke survivors, out of 15 million victims of stroke, face residual difficulties. However, PSCI’s are usually neglected due to more prominent physical symptoms covering the stroke.^5^ Studies have also reported that recovery in PSCI is a potential determinant of improvement in other clinical consequences of stroke including motor and emotional dysfunctions whereas presence of PSCI is highly associated with the possibility of stroke recurrence.^6^

The assessment of PSCI is therefore crucial which not only plays its role in the process of diagnosis but neuropsychological evaluations are utilized to develop an effective treatment and rehabilitation plan.^7^ Most of the existing definitions of post-stroke cognitive disorders are centered on the notions of cognitive decline and dementia. However, the current study defines cognitive impairments in context of decline in cognitive abilities rather than taking them as impairments. Cognition may be considered comprising of five primary domains: attention, memory, executive functions, language and visuospatial skills.^8^

Though there are many widely used neuropsychological screening tools for assessing executive functions in clinical practice today, however, most of these instruments were not developed with the purpose of assessing individuals with language and motor complexities.^9^ The detailed analysis of commonly used neuropsychological test batteries revealed that there is no brief inventory that could cater a wide variety of cognitive impairments. Since majority of the Western scales including Mini Mental Status Examination (MMSE), Montreal Cognitive Assessment (MoCA), Cognistat and others, mainly focus on verbal skills or place demands on linguistic processing (left hemisphere domain) neglecting the nonlinguistic cognitive skills of right hemisphere. Therefore, it could be said that such scales are not applicable for aphasia and also ignore the deficits of right hemisphere lesion such as hemi neglect. This is one of the reasons that right hemisphere lesion remain undetected and favors the notion of left hemisphere stroke being more prevalent.^10^ Most importantly, almost all of the current screening tools, for stroke, require academic exposure (e.g. copying some figure, comprehension or reading tasks) offering less applicability in our culture due to low literacy rate.

Therefore, the purpose of the study was to develop an instrument to address these issues and most importantly to enhance the efficiency and sensitivity of tool with respect to our culture. N-CABS was designed while keeping in consideration the socio demographic variables and possible motor and language difficulties among stroke patients. The difficulty level of tasks was intended to keep in accordance with educational level of Pakistani individuals to make tool sensitive in assessing PSCI. Furthermore, another purpose was to give equal weightage to linguistic and non-linguistics task unlike traditional neuropsychological test batteries. In tis study the goals were (1) to develop N-CABS (2) to identify cut points (3) to compare the performance of N-CABS with MoCA (most widely used tool to assess PSCI) and (4) to validate N-CBAS in an independent sample against a reference standard for cognitive impairment.

## METHODS

The current neuropsychological test battery was developed following four main phases including; Phase 1 (Identification of the types of cognitive impairments after stroke), Phase 2 (Selection and collation of subtests assessing specified PSCI), Phase-3 (Validation of subtests assessing specified PSCI) and Phase-4 (Main study was carried out to assess the pattern of PSCI among patients and to develop psychometric properties).

The most initial phase of research focuses on the determination of types of PSCI which involved review of the existing literature concerning PSCI,^8^,^11^,^12^ discussion with patients and professionals having clinical experience in the diagnosis of stroke. After consulting these sources seven most prevalent cognitive impairments were finalized including orientation, attention, naming, reasoning, arithmetic, new learning/memory and spatial neglect. Following the selection of cognitive impairments, a panel discussion with three neurologists and three neuropsychologists/psychologists was arranged to finalize standardized subtests assessing above mentioned seven cognitive impairments. During selection process, it was taken care to come up with a balanced screening battery equally assessing both verbal and visual cognitive abilities. The final protocol comprised of nine standardized subtests assessing cognitive impairments including; Orientation for temporal orientation, Digit span for verbal attention, Arithmetic for mathematical manipulation, Similarities for abstract reasoning, Naming for word finding difficulty or semantic memory impairment, Paired Associate Learning Test for verbal memory, Bell’s Test for spatial neglect and visual attention, Block Design for spatial abilities and Non-Verbal Learning Test (NVLT) for visual memory. The summary of abilities assessed by subtests and the region of brain associated with those abilities are illustrated in Table-I.

**Table-I:** Subtests, abilities assess by subtests and location of brain associated with abilities.

Subtests	Abilities	Location
Orientation	Orientation to time, place and person	Temporal disorientation
Attention Span	To assess attention & concentration	Digit Forward (Left frontal lobe) Digit Backward (Right frontal lobe)
Paired Associate Learning Test (PALT)	Memory & new learning	Left Temporal Lobe
Naming	Semantic memory &Visuoperceptual skills	Primary Visual Cortex/ Frontal Lobe/Parietal Lobe
Similarities	Pre-morbid intelligence/reasoning abilities	Left Temporal/Frontal Lobe
Arithmetic	Mathematical operations	Frontal Lobe
Bell’s test	Hemi neglect	Right Parietal Lobe
Block design	Visuospatial ability	Parietal Lobe
Non-Verbal Learning Test (NVLT)	Visual Memory	Temporal Lobe

After the selection of subtests a try out phase was conducted to shortlist items from each complex subtest (Digit Span, Paired Associate Learning Test, Block Design, Non-Verbal Learning Test) on the basis of difficulty level. In order to calculate the difficulty level of items of subtests full subtests were administered on 30 participants. Furthermore, during this phase Naming subtest and Similarities subtest were also developed by selecting three most culturally common animals’ pictures as suggested by a sample of 30 participants during the phase of pilot study. The collated version of battery was then sent for expert validation to three of the professionals having expertise in neurological testing. All corrections were incorporated as suggested by the experts before moving to the main phase of study. Following these phases the neuro-cognitive test battery was finalized.

The current study involved 121 participants divided into clinical (stroke patients) and non-clinical groups. The clinical group comprised of 61 participants whereas 60 participants were recruited in non-clinical group. The patients with unilateral stroke were recruited from the stroke ward in Neurology Departments of various government (n=4) and private hospitals (n=6) using purposive sampling. Inclusion criteria of the selection of participants in clinical group include; having a first unilateral stroke, age over 30 years, minimum education till primary and adequate post-stroke fluency in Urdu. Exclusion criteria include bilateral hemisphere, convergent conditions and disorders that might affect any cognitive skills under study, traumatic brain injury, infratentorial lesions, or patients with right hemispheric lesions who were assessed to have aphasia (crossed aphasia).

The healthy matched controls were mostly relatives of patients or employees working in institutions where data was collected. All healthy participants were initially examined by physician for general health and only those participants were included having no motor, intellectual, neurological or sensory deficits or psychiatric disorders. Both groups were matched on demographic variables of age (over 30 years), education (primary or above) and occupation.

### Measures

The subtests selected for the purpose of screening of PSCI were taken from standardized batteries Wechsler Intelligence Scale for Children,^13^ Wechsler Memory Scale,^14^ Bell’s Test^15^ and Non-Verbal Learning Test.^16^ The subtests taken from WISC include: Mental Arithmetic, Digit Span and Block Design whereas subtests chosen from WMS include Orientation and Paired Associate Learning Test (Verbal). The already Urdu translated versions of verbal tests were employed. The same scoring criteria of standardized subtests was used (as suggested in manuals of respective sub-tests) whereas the number of items within some subtests such as Digit Span, PALT, Block Design and Arithmetic were reduced to make battery brief. In order to achieve this purpose the reduction was made on the basis of calculated difficulty level by administering those subtests on 30 participants. A small protocol was also devised to gain information about patient’s cognitive functioning from his/her caregivers which comprised of nine unstructured questions. Other important demographic variables were also assessed such as age, gender, education, occupation, onset, severity and type of stroke. The mode of administration was paper pencil based and the average time of test administration was 10 minutes.

### Ethical Consideration

The study was conducted after getting approval from the ethical committee of Institute of Clinical Psychology. The researcher took verbal informed consent by participants and provided them sufficient information regarding the purpose of the current study. The researcher also ensured the confidentiality of each participant and informed them about their right of leaving the study at any time they want.

### Analysis

The data was analyzed using Statistical Package for Social Science, 21 version (SPSS 21). Analyses comprised of exploring descriptive information through descriptive statistics, exploratory factor analyses (EFA) to establish psychometric properties. The independent sample t-test was used to explore group differences whereas Pearson- product correlation was employed for assessing existing correlation between variables.

## RESULTS

The participants of the study involved both clinical and non-clinical individuals who were matched on various demographic variables (including; age, education and occupation) whereas the manipulated variable of interest was the presence of stroke which distinguished both groups. The frequency distribution of all significant socio demographic variables including age, gender, education, and occupation are provided in Table-II. The total sample comprised of 121 participants among whom 61(51%) belong to clinical group whereas remaining 60 (49%) participants belong to non-clinical group with 42% of all participants falling in the age category of 30 to 40 years whereas 70% fell between 41 to 65 years of age. The frequency distribution of gender revealed that the clinical group comprised of 28 (46%) women and 33 (54%) men whereas non-clinical group included 35 (58%) women and 25 (42%) men. The qualification of 24 (41%) patients and 34 (58%) normal individuals was ranged between primary to intermediate whereas 63 (52%) participants including 30 (49%) patients and 33 (55%) normal individuals were educated till bachelors or masters. The frequency distribution of occupation elucidated that among 37 (31%) non-working (including housewives and unemployed) participants 17 (28%) were patients and 20 (33%) were normal individual. However, 29 (28%) patients and 19 (32%) normal individuals were having occupations related to office job or teaching whereas 15 (25%) patients and 21 (35%) normal individuals were involved in occupations related to construction or requiring motor abilities.

**Table-II:** Frequency distribution and percentage of demographic characteristics of participants.

Variables	Clinical f (%)	Non-clinical f (%)	Total f (%)
***Gender***			
Men	33(54)	25(42)	61(50)
Women	28(46)	35(58)	60(50)
Total	61(51)	60(49)	121(100)
***Age Category***			
Early Adulthood (30-40)	27(22)	24(20)	51(42)
Middle Adulthood (41-65)	34(28)	36(30)	70(58)
***Education***			
Primary and College	34(58)	24(41)	58(36)
Graduate and Above	30(49)	33(55)	63(39)
***Occupation***			
Non-working	17 (28)	20 (33)	37 (30)
Teaching/Office Job	29 (28)	19 (32)	48 (40)
Engineering / Occupations related to motor abilities / Business	15 (25)	21 (35)	36 (30)

To validate NCABS Construct validity was opted which was done using Exploratory Factor Analysis (EFA) through Principal Component Factor Analysis (PCFA). Initially, in order to determine factors of N-CABS, the adequacy of EFA through PCFA had been assessed through Kaiser-Meyer-Olkin (KMO) measure of sampling adequacy and Bartlett’s Test of Sphercity. The value of KMO was 0.79 which was higher than recommended value of 0.6 whereas Bartlett’s Test of Sphercity was also significant (χ^2^ (36) = 250.20, *p* < .005) strongly suggesting EFA. The analysis of diagonals of the anti-image correlation matrix support the inclusion of all 9 items in factor analysis as values of items found to be above 0.5. Furthermore, the output of communalities revealed the values of communality of all variables to be higher than 0.4, further confirming common variance shared by all items. All these indicators highly recommended EFA to be conducted on all 9 items.

**Fig.1:**
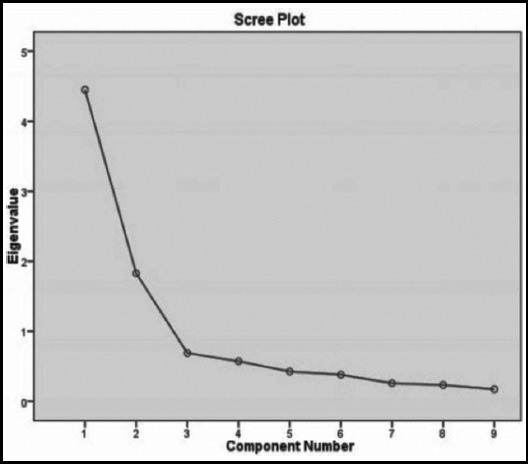
Scree Plot showing two-factor solution with Eigenvalues greater than 1 001, ns= not significant

The initial Eigenvalues as shown in scree plot (Fig.1) obtained through EFA using PCFA and Varimax Rotation employed on 9 sub-tests yielded a two factor solution with first factor explaining 46% of the variance whereas second factor explaining 63% of the variance. The minimum cut off value of factor loadings of items for this study was 0.65 or greater as sample size was less than 100.^17^ The analysis revealed that six sub-tests were loaded on first factor whereas remaining three sub-tests were loaded on second factor. All subtests exhibited loadings of value greater than 0.65 except for orientation which was then excluded. PCFA of the remaining 8 items was again employed using Varimax rotation. This resulted in best defined factor structure with only one item (Bell’s test) having factor loading 0.687 while all others exhibiting factor loading values of 0.70 or above (Table-III). The exclusion of Orientation test not only improved total variance explained by both factors from 63% to 67% (variance explained by factor 1 being 51% and factor 2 being 68%) (Table-IV) but also cause an increase in KMO from 0.79 to 0.83 (χ^2^ (28) = 234.22, *p* < 0.005).

**Table-III:** Factor structure of 9 sub-tests of N-CABS with Varimax Rotation.

Item	Factor 1	Factor 2
Reasoning	0.85	
Arithmetic	0.84	
Naming	0.82	
Paired Associate Learning Test	0.76	
Attention	0.72	
Block Design		0.88
Non-Vebal Learning Test		0.83
Bell’s Test		0.68
		

***Note:*** Factor loadings >0.65 are boldfaced

**Table-IV:** Eigenvalues and Variance Explained by Two Factors of N-CABS.

Factors	Eigenvalues	% of Variance	% of Total Variance
1	4.16	51.99	51.99
2	1.35	68.82	16.83

Furthermore, a descriptive label was given to each respective factor based on the resemblance among subtests categorize under these two factors. The first factor being loaded with five subtests requiring verbal abilities including Attention, Paired Associate Learning Test (PALT), Naming, Reasoning and Arithmetic was named as Verbal Cognitive Abilities (Ver.CA). On the other hand, the second factor being loaded with three subtests involving visual abilities including Bell’s Test, Block Design and Non-Verbal Learning Test (NVLT) was named as Visual Cognitive Abilities (Vis.CA).

Other psychometric properties of N-CABS were established using reliability measures (test retest reliability and internal consistency through inter item correlation) and validity measures (concurrent validity and discriminant validity). The test retest reliability was determined by re-administering N-CABS on 10 patients after duration of one week. The Cronbach alpha of N-CABS found out to be 0.92 with a robust correlation (^***^p<0.001) between both administrations suggesting N-CABS a reliable measure. The internal consistency was established through inter item correlation, the findings of which are presented in Table-V. The output revealed that subtests/items of Ver.CA (Factor 1) including Attention and PALT were significantly correlated with all the items of both factors (Ver. CA and Vis. CA) whereas Naming, Reasoning and Arithmetic were majorly correlated with subtests of Factor 1 only. Similarly, inter item correlation within Vis. CA found to be significantly positive. However, inter item correlation between factors revealed to be negative. This shows that a participant performing well on verbal related skills may likely to perform low on visual related skills.

**Table-V:** Summary of Inter-Item Correlation, Mean and Standard Deviations between Items of Ver.CA and Vis.CA.

Variables	Attention	PALT	Naming	Reasoning	Arithmetic	Bell’s Test	Block Design	NVLT
Attention	---	0.57***	0.62***	0.68***	0.57***	0.31***	0.25**	0.22**
PALT	---	---	0.71***	0.71***	0.70***	0.19*	0.22**	0.28**
Naming	---	---		0.71***	0.78***	0.09^ns^	0.10^ns^	0.89***
Reasoning	---	---	---		0.71***	0.15^ns^	0.29**	0.29**
Arithmetic	---	---	---	---		0.15^ns^	0.17^ns^	0.20**
Bell’s test	---	---	---	---	---		0.55***	0.48***
Block Design	---	---	---	---	---	---	---	0.60***
NVLT	---	---	---	---	---	---	---	---
M (SD)	9.65 (2.47)	13.70 (5.19)	2.70 (0.60)	2.59 (0.64)	2.50 (0.86)	33.56 (2.33)	8.0 (3.53)	10.85 (4.12)

***Note:*** df= 120,

* p<0.05,

** p<0.01,

*** p<0.001, ns= not significant.

Further validation of N-CABS was done using two measures of validity, including; concurrent validity and discriminant validity. The concurrent validity was established by administering another psychometrically strong brief battery measuring cognitive impairements [Montreal Cognitive Assessment Battery (MoCA)] along with N-CABS on 16 patients. The findings of concurrent validity suggested that Cronbach alphas for 13 MoCA items and 8 N-CABS items came out to be 0.82 (^***^p<0.001) between both measures suggesting N-CABS a valid measure for assessing cognitive impairments. Moreover, discriminant validity was conducted using *known group validity* to examine how well NCABS discriminates between group of patients and healthy controls. In order to assess group differences on N-CABS independent sample t-test was carried out. The figures in the Table-VI revealed significant group differences for clinical and non-clinical participants not only across the subtests of N-CABS but also on the total N-CABS. The *p* value across all the subtests and total N-CABS found out to be greater than 0.001^***^ alpha level. Therefore, the findings suggest a substantial presence of PSCI among patients of stroke and also N-CABS was able to successfully discriminate those clinical group and non-clinical group on the basis of participants’ performances.

**Table-VI:** Independent sample t-test for group differences across items of N-CABS and total of N-CABS.

Subtests	Group	M	SD	t	p<
Orientation	Clinical	4.12	1.11	6.18	0.001***
	Non-Clinical	5	0		
Attention	Clinical	7.82	2.25	9.82	0.001***
	Non-Clinical	11.20	1.31		
PALT	Clinical	9.98	4.59	9.28	0.001***
	Non-Clinical	16.87	3.19		
Naming	Clinical	2.35	0.74	6.75	0.001***
	Non-Clinical	3	0		
Reasoning	Clinical	2.14	0.69	9.27	0.001***
	Non-Clinical	2.98	0.13		
Arithmetic	Clinical	1.94	1.01	7.74	0.001***
	Non-Clinical	2.97	0.18		
Bell’s Test	Clinical	32.04	2.72	7.89	0.001***
	Non-Clinical	34.85	0.44		
Block Design	Clinical	6.12	3.21	5.94	0.001***
	Non-Clinical	9.60	2.96		
NVLT	Clinical	8.82	3.65	5.33	0.001***
	Non-Clinical	12.57	3.71		
N-CABS	Clinical	73.29	9.39	15.24	0.001***
	Non-Clinical	98.12	7.78		

***Note:*** M= Mean, SD= Standard Deviation,

* p<0.05,

** p<0.01,

*** p<0.001, ns= not significant

## DISCUSSION

From apoplexy to cerebrovascular accidents, stroke remained associated with devastating consequences since centuries. However, the major dilemma is that despite its shattering consequences the management of this multidisciplinary disease is confined to only one discipline in countries like Pakistan. It is also well-established that improvement in cognitive status positively affects all other affected areas including motor and emotional dysfunctions, therefore, cognitive functioning is considered foremost importance when it comes to the rehabilitation of stroke survivors.^6^,^18^ Keeping in view the current tragic situation regarding the assessment and management of cerebrovascular accidents in Pakistan the current research was planned to take an initiative by initially focusing on the sound assessment of Post Stroke Cognitive Impairments (PSCI).

The initial factor analysis classified N-CABS into two factors; first one containing items related to verbal skills (Verbal Cognitive Abilities; Ver.CA) whereas the second one containing items related to visual skills (Visual Cognitive Abilities; Vis.CA). The division of subtests into two factors was ideal and in accordance with other renowned cognitive assessment batteries like Montreal Cognitive Assessment (MoCA),^19^ Mini-Mental State Examination (MMSE)^20^ and Cognistat.^21^ The study succeeded in developing and validating a cognitive assessment tool that is quick and simple, culturally relevant, requires minimal motor and verbal skills and is unbiased towards patient’s education. To enhance its utility, simple administration and scoring system are established. The psychometric analysis of N-CABS suggested it to be equivalent or superior to widely used MoCA. Therefore, in particular N-CABS may be preferred over other widely used tools in frail individuals with motor/verbal difficulties or having low literacy level and exposure to paper pencil or other relevant tasks.

N-CABS has potential advantages over other neuropsychological screening tools like MMSE, MoCA or Cognistat. As it includes tasks which are more relevant to our culture, for example, in Naming subtest all animals are more common unlike animals used in MoCA. In the same way, these instruments require fine motor skills in some items (cube or clock drawing) limiting its use with individuals with motor deficits in stroke.^21^ Most of these instruments do not emphasize on the assessment and identification of hemi-neglect which is common in right hemisphere stroke, hence, proving less efficient in detecting right hemisphere strokes.^22^,^23^ Furthermore, as problem of low literacy is very common in Pakistan and one of the purposes of developing N-CABS was to an instrument with most generalizability in our culture. N-CABS found to have equivalent or superior performance in comparison of MoCA across all levels of education as it does not include items biased against persons with low education. For example, items like clock drawing, repeating “no ifs ands or buts,” writing a sentence, naming the season, or performing serial 7s were not included.^19^,^20^,^21^ Last but not the least, N-CABS is designed in a way that it includes items of all Verbal correspondent Visual cognitive abilities putting equal emphasis on verbal versus visual abilities to enhance its sensitivity towards PSCI.

The study favored the presence of PSCI among stroke survivors which were rigorously detected by N-CABS. The findings of the current study supported a significant prevalence of PSCI among stroke patients. The results elucidated that approximately 85% of the patients were detected to have PSCI. In Pakistan, very few studies exist determining PSCIs among stroke survivors. However, one of the studies aimed at assessing the prevalence of PSCI found that approximately 26% of the patients were having PSCI 24 whereas another study reported 29% of the patients with mild PSCI, 57% with moderate PSCI and 14% with severe PSCI.^25^ Moreover, the significant variation among the prevalence of PSCI can also be explained by the assessment measures used to screen PSCI, for example, in some studies the prevalence of PSCI also reaches to 96% when assessed with comprehensive neuropsychological test batteries.^26^

## CONCLUSION

The basic purpose of this study was to evaluate a clinically and culturally sound neuropsychological test battery to assess cognitive impairments among stroke patients. Overall, N-CABS was found to be a reliable screening tool to assess six cognitive impairments including Attention, Memory, Naming, Arithmetic, Reasoning and Visual Neglect. It has several advantages over other bedside cognitive tests commonly used to detect PSCI as it is inexpensive, less time consuming and more culturally relevant. In future, a more detailed clinical diagnosis of etiology, volume and site of the lesions could be taken into account to control confounding in the results and get a clearer picture of the pattern of cognitive dysfunctioning. The present study was not able to develop extensive norms due to small sample size of patients, therefore, follow up studies should be planned on norm development of N-CABS. Lastly, the scope of current research was limited to cognitive functioning, future researches should expand the scope to emotional, psychological and motor dysfunctions.

### Note

The current research has been done as a part of graduation project. Therefore, this research received no specific grant from any funding agency in the public, commercial, or not-for-profit sectors. There are no conflicts of interest to disclose.

### Author’s Contribution

**SNM** conceived and designed study, did literature search, collected and analyzed data, did manuscript writing and editing.

**UB** helped in conceptualization of study, statistical analysis and revision of initial and final manuscript.
